# Early Clinical Outcomes of Intensity Modulated Radiation Therapy/Intensity Modulated Proton Therapy Combination in Comparison with Intensity Modulated Radiation Therapy Alone in Oropharynx Cancer Patients

**DOI:** 10.3390/cancers13071549

**Published:** 2021-03-27

**Authors:** Han Gyul Yoon, Yong Chan Ahn, Dongryul Oh, Jae Myoung Noh, Seung Gyu Park, Heerim Nam, Sang Gyu Ju, Dongyeol Kwon, Seyjoon Park

**Affiliations:** 1 Samsung Medical Center, Department of Radiation Oncology, Sungkyunkwan University School of Medicine, Seoul 06351, Korea; hangyul.yoon@samsung.com (H.G.Y.); dongryuloh@skku.edu (D.O.); rodrno@skku.edu (J.M.N.); sg.ju@samsung.com (S.G.J.); dy82.kwon@samsung.com (D.K.); seyjoon@yuhs.ac (S.P.); 2 Keimyung University Dongsan Medical Center, Department of Radiation Oncology, Keimyung University School of Medicine, Daegu 42601, Korea; psk818@dsmc.or.kr; 3 Department of Radiation Oncology, Kangbuk Samsung Hospital, Sungkyunkwan University School of Medicine, Seoul 03181, Korea; heerim.nam@skku.edu; 4 Yonsei Cancer Center, Department of Radiation Oncology, Yonsei University College of Medicine, Seoul 03722, Korea

**Keywords:** acute toxicity, oropharyngeal cancer, proton beam therapy, radiation therapy, survival

## Abstract

**Simple Summary:**

Intensity-modulated proton therapy (IMPT) is expected to reduce toxicity more effectively than intensity-modulated radiation therapy (IMRT) in treating oropharynx cancer (OPC) patients. Because of long waiting before starting IMPT, authors began IMRT first and then determined whether to continue IMRT or to switch into IMPT at time of adaptive re-plan, based on the rival plan comparison in 148 OPC patients. Early clinical outcomes were analyzed and compared between IMRT alone and IMRT/IMPT combination groups through propensity score matching method. We found that, with comparable oncologic outcomes, more favorable acute toxicity profiles (mucositis and need for analgesic use) were achieved following IMRT/IMPT combination than IMRT alone.

**Abstract:**

Purpose: To report the early clinical outcomes of combining intensity-modulated radiation therapy (IMRT) and intensity-modulated proton therapy (IMPT) in comparison with IMRT alone in treating oropharynx cancer (OPC) patients. Materials and Methods: The medical records of 148 OPC patients who underwent definitive radiotherapy (RT) with concurrent systemic therapy, from January 2016 till December 2019 at Samsung Medical Center, were retrospectively reviewed. During the 5.5 weeks’ RT course, the initial 16 (or 18) fractions were delivered by IMRT in all patients, and the subsequent 12 (or 10) fractions were either by IMRT in 81 patients (IMRT only) or by IMPT in 67 (IMRT/IMPT combination), respectively, based on comparison of adaptive re-plan profiles and availability of equipment. Propensity-score matching (PSM) was done on 76 patients (38 from each group) for comparative analyses. Results: With the median follow-up of 24.7 months, there was no significant difference in overall survival and progression free survival between groups, both before and after PSM. Before PSM, the IMRT/IMPT combination group experienced grade ≥ 3 acute toxicities less frequently: mucositis in 37.0% and 13.4% (*p* < 0.001); and analgesic quantification algorithm (AQA) in 37.0% and 19.4% (*p* = 0.019), respectively. The same trends were observed after PSM: mucositis in 39.5% and 15.8% (*p* = 0.021); and AQA in 47.4% and 21.1% (*p* = 0.016), respectively. In multivariate logistic regression, grade ≥ 3 mucositis was significantly less frequent in the IMRT/IMPT combination group, both before and after PSM (*p* = 0.027 and 0.024, respectively). AQA score ≥ 3 was also less frequent in the IMRT/IMPT combination group, both before and after PSM (*p* = 0.085 and 0.018, respectively). Conclusions: In treating the OPC patients, with comparable early oncologic outcomes, more favorable acute toxicity profiles were achieved following IMRT/IMPT combination than IMRT alone.

## 1. Introduction

Radiation therapy (RT) has the capability of organ preservation, and plays a key role, with or without chemotherapy, in managing oropharyngeal cancer (OPC) patients with non-metastatic disease [1,2,3]. With technical advancements, intensity modulated radiation therapy (IMRT), when compared to the traditional 2- or 3-dimensional RT techniques, has enabled high enough radiation dose delivery to the targets at reduced risks of severe acute and delayed side effects. Although IMRT has currently become the most popular and recommended RT technique in treating most head and neck cancer (HNC) types [4,5], a significant proportion of HNC patients still suffer from annoying side effects and lowered quality of life during and following high dose RT [6,7,8,9].

Proton beam therapy (PBT), by virtue of the physical property of the Bragg-Peak phenomenon, can generate a more advantageous dose distribution profile than photon-based RT techniques, including IMRT, and has been in clinical use in treating several cancer types including HNC [10,11,12,13]. Nevertheless, more clinical evidence is needed to confirm whether the physical advantage of PBT can genuinely lead to better therapeutic outcomes in a real-world practice setting. Considering the substantial costs and resources needed for installation and operation of PBT facilities, answering this issue seems even more important.

With these theoretic backgrounds, we intended to apply PBT in treating HNC patients since December 2015, when our PBT facility began its operation [14]. However, the average waiting time interval before starting PBT after a therapeutic decision has been made is about 4~6 weeks, due to limited PBT resources when compared to the clinical demands. It has been well addressed, however, that long waiting times before treatment initiation in treating HNC patients could result in significantly unfavorable oncologic outcomes [15,16]. Xiao et al. demonstrated that the detrimental effects of increased time to treatment initiation were mediated by tumor progression during the waiting time [17], which was demonstrable by comparing the initial clinical stages and the surgical stages. They found that even 1 week’s delay could be detrimental and suggested timely intervention within the first 4 weeks. Although induction chemotherapy before definitive local treatment could also be considered to bridge the gap, which, however, might increase the treatment-related morbidity risk and the care cost without significant clinical benefit [18].

In order to overcome this long waiting before treatment initiation, we developed our strategy to begin the RT course by IMRT (helical Tomotherapy, HT) and then to determine whether to continue IMRT or to switch into intensity modulated PBT (IMPT), based on the rival re-plan comparison, which corresponds to our adaptive re-plan policy. We previously reported the early clinical outcomes and acute toxicity profiles following IMRT only and IMRT/IMPT combination in treating nasopharynx cancer (NPC) patients [19], and now report our experience in treating OPC patients.

## 2. Results

### 2.1. Patients’ Characteristics

The characteristics of all patients and 72 matched patients based on the propensity scores (36 in each group) are summarized in Table 1. The median age of all patients was 60 years (range, 38~76 years), and the majority was male (137 patients, 92.6%). Among all patients, most characteristics were similarly distributed between groups, but the patients in the IMRT/IMPT combination group more frequently had lower T stage (*p* = 0.025) and received unilateral neck irradiation (*p* < 0.001), respectively. Among 72 matched patients, however, all characteristics distributed similarly between groups.

### 2.2. Oncologic Outcomes

**Figure 1:**
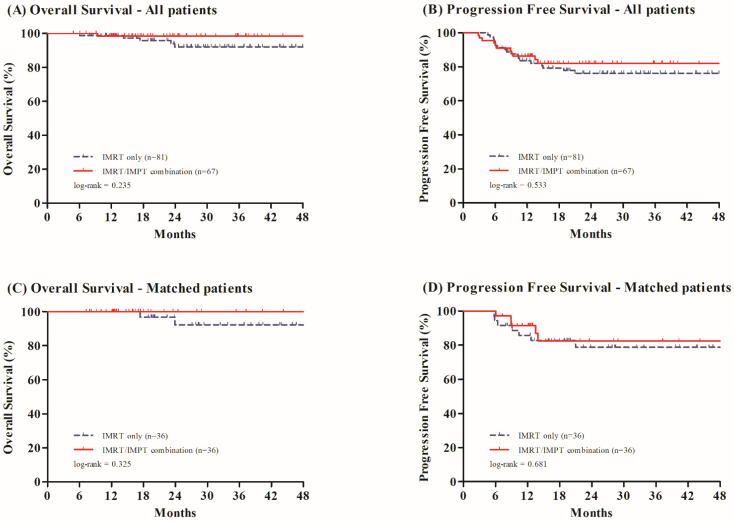
Overall survival and progression free survival among all patients (**A**,**B**) and matched patients (**C**,**D**) according to treatment group. IMRT, Intensity-Modulated Radiation Therapy; IMPT, Intensity-Modulated Proton Therapy.

The tumor response was excellent and the rates of overall and complete response, evaluated at 4 months of RT completion, were 99.3% and 85.1%, respectively. With the median follow-up of 24.7 months (range, 4.9~54.8 months), six patients (4.1%) succumbed to death while 28 (18.9%) experienced treatment failures. The failure sites were locoregional in 14 patients (9.5%), distant in 13 (8.8%), and combined locoregional and distant in one (0.7%), respectively. There were no significant differences of overall survival (OS) and progression-free survival (PFS) profiles between groups, both before and after propensity score matching (PSM) (Figure 1). Among all patients, the 2-year OS rates were 92.1% and 98.4% in the IMRT only and IMRT/IMPT combination groups (*p* = 0.235), and the 2-year PFS rates were 76.2% and 82.0% in the IMRT only and IMRT/IMPT combination groups (*p* = 0.533), respectively. The corresponding figures among the matched patients were 92.4% and 100.0% (*p* = 0.325), and 78.8% and 82.4% (*p* = 0.681), respectively.

### 2.3. Acute Toxicity Profiles

**Figure 2:**
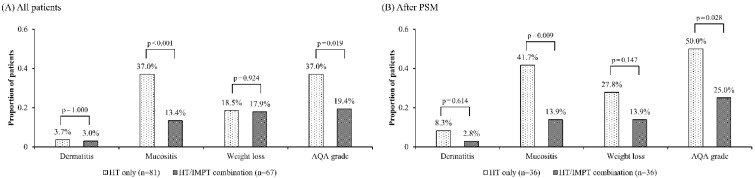
Grade 3 or higher toxicity distribution by treatment group among all patients (**A**), and matched patients (**B**). PSM, Propensity Score Matching; IMRT, Intensity-Modulated Radiation Therapy; IMPT, Intensity-Modulated Proton Therapy.

Table 2 and Figure 2 summarize the acute toxicity profiles. Among all patients, grade ≥ 3 dermatitis, mucositis, weight loss, and Analgesic Quantification Algorithm (AQA) score ≥ 3 (defined as need for strong opioids) occurred in five (3.4%), 39 (28.5%), 27 (18.2%) and 43 (29.1%), respectively (Table 2). Three patients underwent gastrostomy tube feeding during or after RT due to severe oral pain: two in the IMRT only group; and one in the IMRT/IMPT combination group, respectively. The patients in the IMRT/IMPT combination group significantly less frequently experienced grade ≥ 3 mucositis (37.0% vs. 13.4%, *p* < 0.001) and AQA score ≥ 3 (37.0% vs. 19.4%, *p* = 0.019), respectively (Figure 2A). Among the matched patients, the same trends were observed: the frequencies of grade ≥ 3 mucositis were 41.7 % and 13.9% (*p* = 0.009); and those of AQA score ≥ 3 were 50.0% and 25.0% (*p* = 0.028), respectively (Figure 2B).

Univariate and multivariate analyses for grade ≥ 3 mucositis and AQA score ≥ 3 were done, both before and after PSM, respectively (Table 3). The significant factors associated with grade ≥ 3 mucositis among all patients included three variables in univariate analyses: cT3-4 stage (HR = 3.552, 95% CI 1.631~7.737, *p* = 0.001); IMRT only (HR = 3.791, 95% CI 1.646~8.733, *p* = 0.002); and bilateral neck irradiation (HR = 3.723, 95% CI 1.439~9.630, *p* = 0.007), respectively. In multivariate analyses, however, IMRT only remained a significant factor for grade ≥ 3 mucositis (HR = 2.725, 95% CI 1.123~6.615, *p* = 0.027). The factor associated with AQA score ≥ 3 was IMRT only (HR = 2.443, 95% CI 1.148~5.199, *p* = 0.020) in univariate analyses, whose significance declined in multivariate analysis (HR = 2.014, 95% CI 0.907~4.469, *p* = 0.085).

Among the matched cohorts, grade ≥ 3 mucositis was more frequently encountered in the IMRT only group both in univariate (HR = 4.429, 95% CI 1.397~14.039, *p* = 0.011) and multivariate (HR = 4.657, 95% CI 1.353~16.033, *p* = 0.015) analyses, respectively. AQA score ≥ 3 was also more commonly observed in the IMRT only group in univariate (HR = 3.000, 95% CI 1.106~8.138, *p* = 0.031) and multivariate (HR = 2.792, 95% CI 0.993~7.855, *p* = 0.052) analyses, respectively.

## 3. Discussion

Radiation oral mucositis is a very common and unavoidable acute side effect affecting most HNC patients who receive high dose RT. Oral mucositis typically causes oral soreness, swallowing difficulty, decreased oral intake, and subsequent weight loss. Severe oral pain usually necessitates taking painkillers, sometimes narcotics, and the patients may become prone to various adverse effects of the medication. It was reported that RT-related complications, such as oral mucositis, can increase the treatment cost by up to 17,000 USD per patient, and its severity is proportionally associated with the incremental healthcare cost [20,21]. Moreover, modification and/or interruption of the planned RT schedule is occasionally necessary, in order not to compromise the precision of RT and to compensate for the body contour change incurred by these sequence of events in addition to tumor shrinkage itself. Saving the anteriorly located oral cavity mucosal lining could be achieved more effectively in most OPC patients by using IMPT [13].

Adaptive re-plan during the RT course has been highly recommended in order to accommodate the body contour changes in treating most HNC patients [22,23]. The body contour change is usually more significant during the early CCRT course than during the later course. We previously measured the mean tumor volume reduction rates by the time of the re-plan, which were 40.7% in the OPC patients and 41.9% in the NPC patients, respectively [24,25]. These clinically relevant data by the authors strongly endorse the adaptive re-plan strategy, which has long been our institutional policy. We initially wanted to change into the adaptive re-plan usually during the 5th week, when the elective dose to the clinically uninvolved lymphatic regions were delivered. This time frame coincided with the time when we could expect the availability of the proton therapy equipment. For the adaptive RT, we generated two rival plans, one by IMRT and the other by IMPT, and then to determine the subsequent RT modality (whether to continue IMRT or to switch into IMPT), based on the dosimetric profiles in addition to the availability of IMPT. By following these strategies, we could shorten the waiting from 4~6 weeks to a few days, avoid the break during the RT course due to significant and abrupt body contour changes, and determine optimal RT modality on the individual basis. Bhide et al. [26] investigated weekly volume and dosimetric changes (weeks 2~5) during CCRT with IMRT for HNC patients and reported that the most significant CTV changes occurred at week 2. Porceddu et al. [27] prospectively investigated the need for and timing of re-planning in patients with node-positive NPC and OPC, who underwent CCRT. They took daily verification images in 110 patients, which were evaluated on weekly basis, and underwent re-CT to consider the re-plan, should there be a >1 cm difference at any point of the external contour within the treatment area. Twenty-one patients (19.1%) needed to undergo re-CT, and six needed the 2nd re-plan (5.5% among 110 participants and 28.6% among 21 re-CT patients, respectively). They proposed that the re-plan may be considered at the commencement of week 3 for NPC patients and in week 4 of treatment for OPC patients. Castelli at al. [28], using 29 studies on adaptive RT, reviewed the strategies and dosimetric and/or clinical benefits. Although the trend was, more or less, in favor of adaptive RT with respect to local control and toxicity profiles, there was no concrete evidence or consensus on the optimal imaging protocol, frequency and timing of re-planning, which may need to be verified through randomized trials in the future. 

We previously reported that the combination of IMRT and IMPT resulted in more advantageous acute side effect profiles with the equivalent oncologic outcomes [19]. To the best of our knowledge, there have been only a few retrospective studies on OPC patients, which evaluated the causal relationship between IMPT’s dosimetric advantage and RT-related toxicities. Blanchard et al. performed a case-matched analysis comparing IMRT and IMPT for OPC patients and reported reduced rates of gastrostomy tube dependency and severe weight loss (defined as >20% weight loss from the baseline) in the IMPT group [29]. Sio et al., based on 81 OPC patients, demonstrated that the patient-reported symptom burden was lower following IMPT than IMRT [30]. These two studies did neither thorough multivariate analyses nor quantitative measurement of the toxicities, including mucositis and analgesic usage. The current study intended to investigate whether similar effects as those seen in the NPC patients could be obtained in the OPC patients by combining IMRT and IMPT. As described above, the oncologic outcomes of OS and PFS were not different between groups, while the IMRT/IMPT combination, compared with IMRT only, resulted in more favorable acute toxicity profiles in terms of grade ≥ 3 mucositis and AQA score ≥ 3 through the quantitative measurement and multivariate analyses. Our study could have complemented the limitations of the aforementioned studies and, at the same time, supported the consistent finding of improved acute toxicity profiles in treating the OPC patients.

The current study is limited by the uneven distribution of several characteristics between the treatment groups, mainly by virtue of the retrospective nature. We did propensity-score matching and multivariate logistic regression to mitigate this weakness. In addition to the main observations described above, our IMRT/IMPT combination regimen could reduce the direct RT cost up to 28% according to the Korean National Health Insurance plan [31], when compared with 30 fractions’ IMPT only throughout the RT course.

## 4. Materials and Methods

### 4.1. Patients

We retrospectively reviewed the medical records of 177 OPC patients who underwent definitive RT with or without concurrent chemotherapy from January 2016 until December 2019, after approval by our Institutional Review Board (IRB #2018-08-109). After excluding 29 patients, 148 were included in the current study. The reasons for exclusion were unknown human papillomavirus (HPV) status in 15 patients, RT modalities other than IMRT only or IMRT-IMPT combination in 11, and previous history of receiving RT for other head and neck cancers in three, respectively.

All patients underwent the initial evaluation including thorough physical examination, histologic confirmation, and routine diagnostic exams including computed tomography (CT) of the head and neck region and 18F-fluorodeoxyglucose positron emission tomography-computed tomography (PET-CT). For the objective comparison purpose, the clinical stages were assessed according to the 7th edition American Joint Committee on Cancer staging manual, which mainly depended on the anatomic disease extent but not on the HPV status [32].

### 4.2. Treatment Scheme

All patients underwent contrast enhanced CT-based simulation with the tongue positioning device to keep the mouth open. According to our institutional “selective neck irradiation” policy, three target volumes were delineated: gross tumor volume (GTV); high-risk clinical target volume (HR-CTV); and low-risk clinical target volume (LR-CTV), respectively [33]. HR-CTV included the adjacent tissue from the primary GTV by adding 3~5 mm margins in all directions and the immediately adjacent regional lymphatic areas that are 1.0~1.5 cm away from the nodal GTV in the cranio-caudal direction. LR-CTV was individually determined and did not include the clinically uninvolved lymphatics that were two stations away from GTV. The planning target volumes were defined as CTVs plus 0.3 cm margins in all directions, and all target volumes were edited in accordance with the surrounding normal anatomic structures and barriers. This target delineation policy has long been applied to most HNC patients at the authors’ institute, which enabled the clinicians to reduce the elective nodal volume and relevant side effect profiles without increasing the regional failure rate. The same target delineation policy was applied to all patients regardless of the actual RT modality assigned and/or the HPV status. The dose schedules varied along with the study period, which mainly reflected the resource allocation limitation at the authors’ institute. The typical dose schedules to the GTV, HR-CTV, and LR-CTV were 66~68.4 Gy, 60 Gy, and 36 Gy over 30 fractions in 97 patients until January 2018, and 67.2 Gy, 56 Gy, and 32~36 Gy over 28 fractions in 51 patients since February 2018, respectively. The differential dose delivery was possible by combining the simultaneous integrated boost and the adaptive re-plan and shrinking field concept, which eliminated LR-CTV during the later RT course.

**Figure 3:**
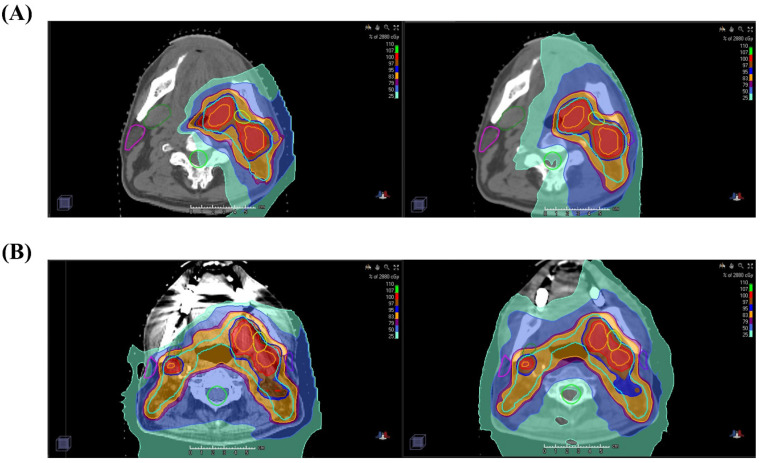
Comparison of dose distribution on axial view between IMRT/IMPT combination (left) and IMRT only (right) in unilateral (**A**) and bilateral (**B**) neck irradiation cases. The areas corresponding to 100%, 97%, 95%, 50% and 25% of the prescribed dose are visualized as red, brown, indigo, blue, and aquamarine colors, respectively. IMRT, Intensity-Modulated Radiation Therapy; IMPT, Intensity-Modulated Proton Therapy.

All patients started RT by IMRT during the early RT course. At the time of adaptive re-plan, two rival plans on each patient, one by IMRT (TomoTherapy^®^, Accuray, Madison, WI, USA) and the other by IMPT (RayStation^®^, RaySearch Laboratories AB, Stockholm, Sweden), were generated under the same policy of target delineation and dose constraints for objective dosimetric comparison (Figure 3). We intended to assign RT modality during the later RT course based on the rival plan comparison. In most patients, saving the oral cavity from high radiation dose was more frequently achieved by the IMPT plan, which, however, sometimes was negated by higher radiation exposure to the spinal cord, the salivary glands, and the skin, depending on the target geometry in relation to these organs at risk. The actual RT modality assignment, however, did not solely depend on dosimetric superiority, but had to be allocated, considering the practical resource limitation and availability. The patients who showed equivalence or superiority by IMRT plan were allocated to IMRT. Meanwhile, those who showed dosimetric superiority by IMPT but should have had a RT break longer than a week for subsequent IMPT were allocated to IMRT in order to avoid the undesirable treatment interruption. Consequently, 81 patients (54.7%) continued to receive IMRT (IMRT only group) and 67 (45.3%) received IMRT + IMPT (IMRT/IMPT combination group), respectively (Table 1).

Along with RT, 139 patients (93.9%) received concurrent chemotherapy during the RT course, while nine underwent RT alone. The intended chemotherapy regimens were 2 cycles of triweekly cisplatin (100 mg/m^2^) in 118 patients (79.7%), 6 cycles of weekly cisplatin (35 mg/m^2^) in nine (6,1%), and cetuximab (400 mg/m^2^ loading dose followed by 5 weekly dose of 250 mg/m^2^) in 12 (8.1%), respectively (Table 1). The vast majority of patients (130, 93.5%) were able to complete the planned chemotherapy cycles, while nine did not because of toxicity. Seven patients among 118 (5.9%) in whom 2 cycles of triweekly cisplatin was planned received only 1 cycle, and two among nine (22.2%) in whom weekly cisplatin were planned received <6 cycles, and all 12 in whom cetuximab was planned were able to complete the intended dose schedule.

### 4.3. Propensity Score Matching

In order to adjust the differences in the baseline characteristics in groups, a PSM method was used. We built a multivariate logistic regression model including the variables with a *p*-value < 0.1 on the Chi-square test or Fisher’s exact and variables that were thought to be possible confounders (age, current smoking, HPV status, clinical T stage, clinical N stage, and bilateral neck irradiation). In order to guarantee the homogeneity, only the patients who receive cisplatin-based chemotherapy and the primary tumor site of tonsil or base of tongue were included at the time of matching. Based on the calculated propensity score, the matching ratio was 1:1 with the caliper set at 0.2. 

### 4.4. Assessment of Acute Side Effects and Response and Follow-Up Schedule after Treatment

The acute toxicity profiles during RT were evaluated at least once a week on each patient by the radiation oncologist in charge: the Common Terminology Criteria for Adverse Events (CTCAE) ver. 4.03 [34] to monitor radiation dermatitis, oral mucositis, and weight loss; and the AQA scoring system to quantify the analgesic usage (Supplementary Table S1) [35].

Response evaluation was done by neck CT taken 1 month after RT completion and PET-CT taken 3 months thereafter. The PET response criteria in solid tumors (PERCIST) was used to assess the early tumor response [36]. Subsequent follow-up evaluations, including neck CT, were regularly scheduled: at every 3~4 months’ interval during the first 2 years; and at every 6 months’ interval thereafter. Locoregional failure was defined as any development of new lesion or progression of preexisting lesion, either within or near the initial disease sites, which were apparent during the regular follow-up evaluation including physical examination and diagnostic imaging studies. Histopathologic confirmation of locoregional failure was tried whenever clinically feasible. 

### 4.5. Statistical Analysis

All statistical analyses were performed using the SPSS software version 24.0 (IBM Corporation, Armonk, NY, USA) and R version 4.0.0 (R Development Core Team, Vienna, Austria, http://www.r-project.org (accessed on 2 June 2020). The OS and PFS rates of the two groups were calculated using the Kaplan–Meier estimate and compared by log-rank tests. To compare the patient characteristics and acute toxicity profiles between the two treatment groups, the Chi-square test or Fisher’s exact test was used for categorical variables while the Student’s t-test was used for continuous variables. Furthermore, multivariate logistic regression was performed in order to identify factors that are independently associated with acute toxicity. Factors with a *p*-value < 0.1 on the univariate analysis or factors considered clinically significant were included in the multivariate analysis, after exclusion of the possible confounding factors.

## 5. Conclusions

In summary, our strategy of combining IMRT and IMPT could avoid undesirable long waiting before treatment initiation, and lead to favorable acute toxicity profiles, at similar oncologic outcomes in treating the OPC patients. Further prospective studies or randomized controlled studies with a larger sample size and longer-term observation including the delayed side effect profiles would be needed.

## Figures and Tables

**Table 1:** Baseline demographic and clinical characteristics.

Variables	All Patients (*n* = 148)			Matched Patients (*n* = 72) *		
	IMRT Only	IMRT/IMPT	*p*-Value	IMRT Only	IMRT/IMPT	*p*-Value
	(*n* = 81)	(*n* = 67)		(*n* = 36)	(*n* = 36)	
**Age**	62.02 ± 8.74 years	59.90 ± 9.62 years	0.161	58.36 ± 6.91 years	58.58 ± 6.03 years	0.885
**Gender**			0.225 ^†^			0.199 ^†^
**Male**	77 (95.1%)	60 (89.6%)		35 (97.2%)	31 (86.1%)	
**Female**	4 (4.9%)	7 (10.4%)		1 (2.8%)	5 (13.9%)	
**ECOG PS**			0.866 ^†^			0.239 ^†^
**0**	4 (4.9%)	5 (7.5%)		3 (8.3%)	-	
**1**	76 (93.8%)	61 (91.0%)		33 (91.7%)	36 (100.0%)	
**2**	1 (1.2%)	1 (1.5%)		-	-	
**Current smoking**			0.433			0.405
**Yes**	24 (29.6%)	16 (23.9%)		29 (80.6%)	26 (72.2%)	
**No**	57 (70.4%)	51 (76.1%)		7 (19.4%)	10 (27.8%)	
**HPV status**			0.889			1
**Positive**	67 (82.7%)	56 (83.6%)		33 (91.7%)	32 (88.9%)	
**Negative**	14 (17.3%)	11 (16.4%)		3 (8.3%)	4 (10.5%)	
**Clinical T stage**			0.025			0.742 ^†^
**T1**	15 (18.5%)	18 (26.9%)		9 (25.0%)	10 (27.8%)	
**T2**	36 (44.4%)	39 (58.2%)		16 (44.4%)	19 (52.8%)	
**T3**	18 (22.2%)	7 (10.4%)		7 (19.4%)	5 (13.9%)	
**T4**	12 (14.8%)	3 (4.5%)		4 (11.1%)	2 (5.6%)	
**Clinical N stage**			0.327 ^†^			1.000 ^†^
**N0**	5 (6.2%)	8 (11.9%)		-	-	
**N1**	10 (12.3%)	10 (14.9%)		5 (13.9%)	4 (11.1%)	
**N2**	61 (75.3%)	48 (71.6%)		30 (83.3%)	31 (86.1%)	
**N3**	5 (6.2%)	1 (1.5%)		1 (2.8%)	1 (2.8%)	
**Staging (AJCC 7th)**			0.519 ^†^			1.000 ^†^
**I**	-	1 (1.5%)		-	-	
**II**	4 (4.9%)	6 (9.0%)		-	-	
**III**	11 (13.6%)	10 (14.9%)		5 (13.9%)	4 (11.1%)	
**IV**	66 (81.5%)	50 (74.6%)		31 (86.1%)	32 (88.9%)	
**Subsite**			0.067 ^†^			0.772
**Tonsil**	55 (67.9%)	53 (79.1%)		29 (80.6%)	28 (77.8%)	
**Base of tongue**	23 (28.4%)	9 (13.4%)		7 (19.4%)	8 (22.2%)	
**Others**	3 (3.7%)	5 (7.5%)		-	-	
**Neck irradiation**			<0.001			0.599
**Unilateral**	15 (18.5%)	35 (52.2%)		9 (25.0%)	11 (30.6%)	
**Bilateral**	66 (81.5%)	32 (47.8%)		27 (75.0%)	25 (69.4%)	
**Concurrent chemotherapy**			0.673 ^†^			1
**Cisplatin**	69 (85.2%)	58 (86.6%)		36 (100.0%)	36 (100.0%)	
**Cetuximab**	5 (6.2%)	7 (10.4%)		-	-	
**No chemotherapy**	7 (8.6%)	2 (3.0%)		-	-	

ECOG PS, Eastern Cooperative Oncology Group Performance Status; IMRT, Intensity-Modulated Radiation Therapy; IMPT, Intensity-Modulated Proton Therapy; HPV, Human Papillomavirus; AJCC, American Joint Committee on Cancer. * Six variables (Age, Current smoking, HPV status, T stage, N stage, and bilateral neck irradiation) were used in the matching process. ^†^ Using Fisher’s exact test.

**Table 2:** Acute toxicity profiles.

Toxicity	All Patients (*n* = 148)			Matched Patients (*n* = 72)		
	IMRT Only (*n* = 81)	IMRT/IMPT (*n* = 67)	*p*-Value	IMRT Only (*n* = 36)	IMRT/IMPT (*n* = 36)	*p*-Value
**Dermatitis**			0.969 ^†^			0.235 ^†^
**Grade 0**	23 (28.4%)	21 (31.3%)		7 (18.4%)	14 (36.8%)	
**Grade 1**	35 (43.2%)	29 (43.3%)		19 (50.0%)	17 (44.7%)	
**Grade 2**	20 (24.7%)	15 (22.4%)		11 (28.9%)	6 (15.8%)	
**Grade 3**	3 (3.7%)	2 (3.0%)		1 (2.6%)	1 (2.6%)	
**Mucositis**			0.009 ^†^			0.012 ^†^
**Grade 0**	-	-		-	-	
**Grade 1**	8 (9.9%)	12 (17.9%)		2 (5.6%)	3 (8.3%)	
**Grade 2**	43 (53.1%)	46 (68.7%)		19 (52.8%)	28 (77.8%)	
**Grade 3**	26 (32.1%)	8 (11.9%)		15 (41.7%)	4 (11.1%)	
**Grade 4**	4 (4.9%)	1 (1.5%)		-	1 (2.8%)	
**Weight loss**			0.245			0.071
**Grade 0**	12 (14.8%)	17 (25.4%)		7 (19.4%)	6 (16.7%)	
**Grade 1**	25 (30.9%)	23 (34.3%)		6 (16.7%)	16 (44.4%)	
**Grade 2**	28 (34.6%)	15 (22.4%)		13 (36.1%)	9 (25.0%)	
**Grade 3**	16 (19.8%)	12 (17.9%)		10 (27.8%)	5 (13.9%)	
**AQA grade**			0.042 ^†^			0.085 ^†^
**Grade 0**	4 (4.9%)	8 (11.9%)		2 (5.6%)	2 (5.6%)	
**Grade 1**	42 (51.9%)	44 (65.7%)		14 (38.9%)	24 (66.7%)	
**Grade 2**	5 (6.2%)	2 (3.0%)		2 (5.6%)	1 (2.8%)	
**Grade 3 or higher**	30 (37.0%)	13 (19.4%)		18 (50.0%)	9 (25.0%)	

IMRT, Intensity-Modulated Radiation Therapy; IMPT, Intensity-Modulated Proton Therapy; AQA, Analgesic Quantification Algorithm. ^†^ Using Fisher’s exact test.

**Table 3:** Univariate and multivariate logistic regression for grade ≥ 3 mucositis and analgesic quantification algorithm score ≥ 3.

Variables *	All Patients (*n* = 148)				Matched Patients (*n* = 72)			
	Univariate Analysis		Multivariate Analysis		Univariate Analysis		Multivariate Analysis	
	OR (95% CI)	*p*-Value	OR (95% CI)	*p*-Value	OR (95% CI)	*p*-Value	OR (95% CI)	*p*-Value
**Grade ≥ 3 Mucositis**								
**Age**	1.030 (0.989–1.072)	0.159	Not included	-	1.084 (0.988–1.188)	0.088	1.090 (0.987–1.204)	0.088
**Clinical T stage**								
**T1-2**	Ref	-	Ref	-	Ref	-	Ref	-
**T3-4**	3.552 (1.631–7.737)	0.001	2.328 (0.980–5.530)	0.056	3.909 (1.254–12.183)	0.019	3.289 (0.870–12.426)	0.079
**Neck irradiation**								
**Unilateral**	Ref	-	Ref	-	Ref	-	Ref	-
**Bilateral**	3.723 (1.439–9.630)	0.007	1.846 (0.629–5.417)	0.264	2.752 (0.708–10.695)	0.144	1.445 (0.304–6.874)	0.643
**RT modality**								
**IMRT/IMPT combination**	Ref	-	Ref	-	Ref	-	Ref	-
**IMRT only**	3.791 (1.646–8.733)	0.002	2.725 (1.123–6.615)	0.027	4.429 (1.397–14.039)	0.011	4.657 (1.353–16.033)	0.015
**Analgesic Quantification Algorithm Score ≥ 3**								
**Clinical T stage**								
**T1-2**	Ref	-	Not included	-	Ref	-	Ref	-
**T3-4**	1.801 (0.841–3.853)	0.13	Not included	-	3.732 (1.266–11.631)	0.02	3.051 (0.896–10.389)	0.074
**Neck irradiation**								
**Unilateral**	Ref	-	Ref	-	Ref	-	Ref	-
**Bilateral**	2.420 (1.052–5.566)	0.038	1.892 (0.784–4.567)	0.156	2.200 (0.695–6.962)	0.18	1.432 (0.401–5.117)	0.581
**RT modality**								
**IMRT/IMPT combination**	Ref	-	Ref	-	Ref	-	Ref	-
**IMRT only**	2.443 (1.148–5.199)	0.02	2.014 (0.907–4.469)	0.085	3.000 (1.106–8.138)	0.031	2.792 (0.993–7.855)	0.052

OR, Odds Ratio; CI, Confidence Interval; RT, Radiotherapy; IMRT, Intensity-Modulated Radiation Therapy; IMPT, Intensity-Modulated Proton Therapy. * Variables with a *p*-value < 0.1 on the univariate analysis of the entire or matched cohort were included in the table.

## Data Availability

The data presented in this study are available on request from the corresponding author. The data are not publicly available due to institutional guidelines.

## Supplementary Materials

The following are available online at https://www.mdpi.com/article/10.3390/cancers13071549/s1. Supplementary Table S1. Analgesic quantification algorithm scoring system.
