# Supplementary material for: Early Clinical Outcomes of Intensity Modulated Radiation Therapy/Intensity Modulated Proton Therapy Combination in Comparison with Intensity Modulated Radiation Therapy Alone in Oropharynx Cancer Patients
Source: Cancers (Basel). 2021 Mar 27;13(7):1549. doi: 10.3390/cancers13071549 (PMC8037748; doi:10.3390/cancers13071549)
Supplement: Supplementary file 1 [file cancers-13-01549-s001.pdf]

Supplementary Table S1. Analgesic quantification algorithm scoring system.

AQA score	Description
**0**	No analgesic
**1**	Non-opioid analgesics
**2**	Weak opioids
**3**	Strong opioids ≤ 75 mg OME per day
**4**	Strong opioids > 75–150 mg OME per day
**5**	Strong opioids > 150–300 mg OME per day
**6**	Strong opioids > 300–600 mg OME per day
**7**	Strong opioids > 600 mg OME per day

AQA, Analgesic Quantification Algorithm; OME, oral morphine equivalent.
